# Comparative effectiveness of post-discharge strategies for hospitalized smokers: study protocol for the Helping HAND 2 randomized controlled trial

**DOI:** 10.1186/s12889-015-1484-0

**Published:** 2015-02-07

**Authors:** Zachary Z Reid, Susan Regan, Jennifer HK Kelley, Joanna M Streck, Thomas Ylioja, Hilary A Tindle, Yuchiao Chang, Douglas E Levy, Elyse R Park, Daniel E Singer, Kelly M Carpenter, Michele Reyen, Nancy A Rigotti

**Affiliations:** Tobacco Research and Treatment Center, Massachusetts General Hospital, Boston, MA USA; Mongan Institute for Health Policy, Massachusetts General Hospital and Partners HealthCare, Boston, MA USA; Division of General Internal Medicine, Medical Service, Massachusetts General Hospital, Boston, MA USA; Department of Medicine, Harvard Medical School, Boston, MA USA; Department of Psychiatry, Harvard Medical School, Boston, MA USA; Department of Psychology, University of Vermont, Burlington, VT USA; School of Social Work, University of Pittsburgh, Pittsburgh, PA USA; Department of Medicine, Vanderbilt Initiatives in Tobacco, Addiction and Lifestyle (VITAL) Center, Vanderbilt University Medical Center, Nashville, TN USA; Alere Wellbeing, Inc., Research Center, Seattle, WA USA

**Keywords:** Smoking cessation, Hospitalization, Pharmacotherapy, Counseling, Interactive voice response, Randomized controlled trial

## Abstract

**Background:**

Smoking cessation interventions for hospitalized smokers are effective in promoting smoking cessation, but only if the tobacco dependence treatment continues after the patient leaves the hospital. Sustaining tobacco dependence treatment after hospital discharge is a challenge for health care systems. Our previous single-site randomized controlled trial demonstrated the effectiveness of an intervention that facilitated the delivery of comprehensive tobacco cessation treatment, including both medication and counseling, after hospital discharge. We subsequently streamlined the intervention model to increase its potential for dissemination. This new model is being tested in a larger multi-site trial with broader eligibility criteria in order to enroll a more representative sample of hospitalized smokers. This paper describes the trial design and contrasts it with the earlier study.

**Methods/Design:**

A 2-arm, 3-site randomized controlled trial is testing the hypothesis that a multi-component Sustained Care intervention is more effective than Standard Care in helping hospitalized cigarette smokers stop smoking after hospital discharge. The trial enrolls adult daily cigarette smokers who are admitted to 1 of 3 participating hospitals in Massachusetts or Pennsylvania. Participants receive the same smoking cessation intervention in the hospital. They are randomly assigned to receive either Standard Care or Sustained Care after hospital discharge. Participants in the Sustained Care arm receive a free 3-month supply of FDA-approved smoking cessation medication and 5 interactive voice response calls that provide tailored motivational messages, medication refills, and access to a live tobacco treatment counselor. Participants in the Standard Care arm receive a smoking cessation medication recommendation and information about community resources. Outcomes are assessed at 1, 3, and 6 months after discharge. The primary outcome is biochemically-validated tobacco abstinence for the past 7 days at 6-month follow-up. Other outcome measures include self-reported tobacco abstinence measures, use of medication and counseling after discharge, hospital readmissions, and program cost-effectiveness.

**Discussion:**

We adapted a proven intervention for hospitalized smokers to enhance its potential for dissemination and are testing it in a multi-site trial. Study enrollment data suggests that the trial achieved the goal of recruiting a broader sample of hospitalized smokers.

**Trial registration:**

Comparative Effectiveness of Post-Discharge Strategies for Hospitalized Smokers (Helping HAND2) NCT01714323. Registered October 22, 2012.

## Background

Cigarette smoking is the leading preventable cause of death in the United States, responsible for nearly 500,000 deaths annually [1]. Stopping smoking increases life expectancy at any age [2]. Over half of smokers attempt to quit each year [3]. Using evidence-based treatment, consisting of pharmacotherapy and counseling support, increases the success of a quit attempt, but only one-third of smokers use any treatment when trying to quit [3].

Each year nearly four million smokers in the United States spend at least one night in a hospital [4]. A hospital admission provides a smoker with a unique opportunity to quit for several reasons. Hospital policy requires smokers to abstain temporarily from tobacco use. If the admission is attributable to a tobacco-related illness, hospitalization may make the risk of tobacco use more personally relevant and increase a smoker’s motivation to quit [5]. Hospital staff can encourage smoking cessation at this teachable moment and guide the smoker to resources to sustain smoking cessation after discharge. Smokers may be given nicotine replacement therapy (NRT) to relieve nicotine withdrawal symptoms during the hospital stay. This provides the smoker personal experience with an effective treatment that he or she may have avoided due to misinformation about its risks [6]. Smokers who use NRT in the hospital are more likely to use it after discharge [7].

Starting a smoking cessation intervention in the hospital increases the odds that a smoker will stop smoking after discharge. Counseling and use of NRT each improve cessation rates, but these interventions have long-lasting effects only if continued for more than a month after hospital discharge [8]. However, many smokers have no plans to sustain tobacco treatment after hospital discharge. Health insurers may not cover the cost of non-prescription NRT that may have been recommended to smokers at discharge [9,10]. Smokers often return home to a household containing other smokers or to an environment filled with other cues to smoke. Consequently, nearly half of all smokers return to smoking within three days of hospital discharge [11].

We previously developed a multi-component intervention to bridge the gap between inpatient and outpatient cessation services by facilitating smokers’ access to the two components of effective tobacco dependence treatment, pharmacotherapy and counseling support, after hospital discharge [12]. To encourage medication use, patients received a free 30-day supply of their choice of smoking cessation pharmacotherapy at discharge, with the option of two free refills. This eliminated patients’ expenditures and avoided the need to visit a pharmacy to obtain medication. In a variety of settings, reducing barriers to obtaining free cessation medications has been shown to increase the use of medications by smokers who are trying to quit [13-19].

The intervention used interactive voice response (IVR) technology to sustain contact with smokers after discharge. IVR is a telephone technology in which a computer detects a voice and touch tones, and responds to callers with a pre-recorded audio script [20]. When applied in the post-discharge setting, automated calls can serve multiple functions. IVR calls provide an efficient method to achieve rapid telephone contact soon after discharge, when patients are at a high risk for relapse. IVR calls remind patients of their plan to quit smoking, provide motivational messages to encourage cessation efforts and promote medication adherence, and facilitate medication refills. IVR calls can also triage patients to live counselors who can provide additional support and encourage adherence to medications by assessing and managing side effects. By using a computer as opposed to a human caller, the IVR system reduces the cost of contacting smokers and can reach patients at their preferred calling times by calling outside of normal business hours. IVR systems have previously been shown to be efficacious in sustaining contact with smokers after discharge. In a pre-post study, Reid and colleagues used an IVR system, which increased 6-month continuous quit rates from 18% to 29% [21].

We tested the effectiveness of our post-discharge tobacco treatment model in the Helping Hospital-initiated Assistance for Nicotine Dependence study (Helping HAND 1), a randomized controlled trial of 397 smokers admitted to one large Boston, MA, hospital. Smokers who received smoking cessation counseling in the hospital and planned to quit smoking after discharge were randomly assigned to the Sustained Care intervention, which provided free medication and IVR follow-up for 3 months, or to Standard Care. Smokers assigned to Sustained Care were more likely to use smoking cessation therapy (both pharmacotherapy and counseling) after hospital discharge and were more likely to achieve the primary outcome measure, biochemically-validated past 7-day tobacco abstinence at 6 month follow-up (26% vs. 15%; RR 1.71, 95% CI, 1.14-2.56) [22].

The current study, Helping HAND 2 (HH2), builds on the Helping HAND 1 (HH1) trial in several ways that aim to test the intervention’s potential when applied to a more diverse patient population and setting (Table 1). To determine whether the HH1 study findings apply to a more diverse population, we broadened study inclusion criteria to include smokers with alcohol and other substance abuse. Although they have a high prevalence of smoking [23], these individuals had been excluded from the HH1 trial because of concern about medical or psychiatric instability. We also prioritized the recruitment of smokers with HIV infection, who also have a high smoking prevalence [24]. Furthermore, we expanded the program from one to three hospitals located in two states and included both academic and community hospitals. Additionally, we streamlined the intervention in order to facilitate its adoption into actual clinical practice. To access a live counselor, participants in the HH1 study used the IVR system to request a separate call back from the hospital counselor at a later time. In contrast, in the HH2 trial, when participants indicate the desire to speak with a counselor during an IVR call, they are immediately transferred during that call to a telephone counseling service. That service is identical to a telephone quitline, a community-based smoking cessation resource that is available free to every U.S. smoker at www.smokefree.gov. Finally, to demonstrate the stability of tobacco abstinence, we extended follow-up to 12-months to assess smoking status of participants who reported uninterrupted abstinence at 6 months after discharge.

**Table 1:** **Major study design differences between Helping HAND 1 and Helping HAND 2**

**Characteristics**	**Helping HAND 1**	**Helping HAND 2**	**Goal of the Change**
**Enrollment**			
Study sites	MGH	MGH, NSMC, UPMC	Increase generalizability
Enrollment criteria	Exclude active alcoholics	Include active alcoholics	Increase generalizability
	Exclude past year substance users (except marijuana)	Substance use allowed unless current admission was for IV drug overdose	
	No suicidal attempt in past year	No suicidal attempt in past 3 months	
Patients with HIV infection	Not prioritized	Prioritized	Increase enrollment of vulnerable population with high prevalence of smoking
**Intervention**			
Counseling after hospital discharge	Hospital tobacco counselor by telephone	Quit Coach by telephone	Streamline access to live counselor by linking IVR call directly to counseling resource using ‘real world’ counseling resource
Nicotine Replacement Therapy after discharge	Refilled and shipped by hospital study staff	NRT refilled through Quit Coach and shipped from Alere warehouse	Streamline medication refill process using ‘real-world’ design
**Follow-up**			
Follow-up calls	1, 3, and 6 months	1, 3 and 6 months (all) 12 months if continuously abstinent at 6-month follow-up	Determine stability of cessation

MGH = Massachusetts General Hospital.

NSMC = North Shore Medical Center.

UPMC = University of Pittsburgh Medical Center.

## Methods

### Study design

HH2 is a multi-center randomized controlled trial testing the hypothesis that the Sustained Care intervention is more effective than Standard Care in helping hospitalized cigarette smokers quit smoking long-term. All participants receive the same smoking cessation intervention in the hospital and are randomly assigned for post-discharge care to one of two study arms. All participants are followed for 6 months; those reporting continuous abstinence at 6 months are followed for an additional 6 months. The primary outcome is biochemically-validated past 7-day tobacco abstinence rates at 6 month follow-up. The study is approved by the Partners Health System Institutional Review Board, the University of Pittsburgh Institutional Review Board, and is registered with the National Institutes of Health Clinical Trials Registry (#NCT01714323).

### Setting

Participants are enrolled from three nonprofit acute care general hospitals in Massachusetts and Pennsylvania. Massachusetts General Hospital (MGH) is a 900-bed urban teaching hospital in Boston, MA, that is affiliated with Harvard Medical School; it had 49,079 admissions in 2013. North Shore Medical Center (NSMC) is a 411-bed suburban community hospital in Salem, MA that admitted 18,428 patients in 2013. The University of Pittsburgh Medical Center (UPMC), consisting of Montefiore and Presbyterian University Hospitals, is a 799-bed urban teaching hospital in Pittsburgh, PA that had 42,122 admissions in 2013.

### Recruitment

Study inclusion and exclusion criteria are listed in Table 2. A multi-step process identifies eligible patients. Clinical staff at each site document every patient’s smoking status in the electronic health record at admission. Each site’s Tobacco Treatment Service (TTS) receives a daily electronic list of all newly-admitted smokers. A certified TTS counselor, whose background is in nursing, social work, or health education, attempts to see each newly-admitted smoker at bedside, usually 24-48 hours after hospital admission. Following a protocol, the counselor assesses nicotine withdrawal symptoms and recommends that most smokers receive NRT for withdrawal symptom relief during hospitalization. Because previous studies have found combination NRT to be more effective than single NRT products [25], the usual recommendation is a combination of NRT products, consisting of the nicotine patch supplemented by a short-acting product (nicotine lozenge, inhaler, or gum) that is used as needed to suppress nicotine withdrawal symptoms.

**Table 2:** **Study inclusion and exclusion criteria**

Inclusion criteria	
•	Admission to a participating hospital
•	Received tobacco cessation counseling for > 5 minutes in hospital
•	Age ≥18 years
•	Current daily smoker (defined as having smoked ≥1 cigarette/day in the past month when smoking as usual)
•	Plan to sustain or initiate a quit attempt immediately after hospital discharge^1^
Exclusion criteria	
•	Non-English speaking
•	Unable to provide informed consent due to serious cognitive impairment or impaired mental status (e.g., current diagnosis of schizophrenia, psychosis, dementia, or severe mental retardation)
•	Life expectancy of <1 year
•	Are admitted to the hospital from a nursing home
•	Admission diagnosis of intravenous drug use overdose
•	Attempted suicide in the past 3 months
•	Pregnant or nursing
•	Do not agree to take tobacco cessation medication home at discharge
•	No mailing address
•	No phone where can be directly reached or unable to communicate by telephone
•	Decline to speak to study staff or have insufficient time before discharge to be enrolled
•	Are not expected to be discharged home or to a rehabilitation facility

^1^Assessed by asking the smoker to endorse one of 4 responses *(‘I will stay quit’, ‘I will try to quit’, ‘I don’t know if I’m going to quit’, ‘I do not plan to quit’*). Only smokers who endorse the first 2 responses are eligible for study enrollment.

The TTS counselor also inquires about patients’ plan regarding their smoking after hospital discharge. If a patient clearly is not motivated to quit, contact time with the TTS counselor is typically less than five minutes. These patients are not referred for study recruitment. Patients who plan to stay quit or try to quit after discharge receive a median of 20 minutes of bedside counseling to create a personalized quit plan. The session includes a specific smoking cessation medication recommendation, tips on how to abstain from smoking after discharge, and information about counseling services available after discharge. At the end of the counseling session, the TTS counselor offers smokers who plan to quit after discharge and meet inclusion criteria information about the study and refers them to research staff for recruitment. Research staff visit the smoker in the hospital to describe the study, assess eligibility, obtain informed consent, collect baseline data, and assign the patient to a study arm. Research staff also prioritize the recruitment of patients with HIV infection on any day when such a patient is referred to them. The participant’s primary care physician is notified about the trial and about any medications prescribed with a note in the electronic health record or by fax.

### Assignment to treatment group

At each study site participants are randomly assigned to either Standard Care or Sustained Care by research staff in a 1:1 fashion using permuted blocks of 8. Randomization is stratified into five groups: four strata were created by crossing daily cigarette consumption (<10 versus ≥10 cigarettes per day) with admitting service (cardiac vs. non cardiac), and a fifth stratum was created for HIV-infected patients. A series of sealed manila envelopes was created for each stratum, with the randomization ID on the outside of the envelope and the study arm assignment inside the envelope. The research assistant informs the participant and initiates the protocol for the assigned study arm. The study is not blinded to participants or to research staff, but enrollment and follow up activities are conducted by research staff members not involved with recruitment.

### Interventions

#### Sustained care

The multi-component Sustained Care intervention facilitates access to two components of effective tobacco dependence treatment, pharmacotherapy and counseling support, following discharge [26]. It uses IVR technology to provide counseling support and medication management after discharge and offers participants up to 90 days of free smoking cessation pharmacotherapy, with a 30-day supply provided in hand at discharge. The intervention closely resembles the Sustained Care intervention of the HH1 trial, differing primarily in how counseling is provided to smokers who request it during an IVR call [12].

#### Interactive voice response (IVR)

**Figure 1:**
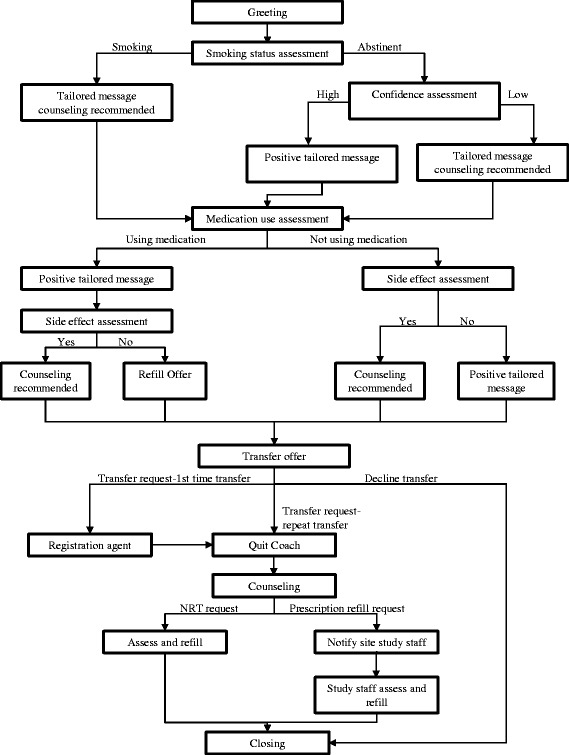
**Sample Interactive Voice Response and Quit Coach design.**

The IVR system provides automated telephone calls at 2, 12, 28, 58, and 88 days after discharge (Figure 1). For each call, the IVR system makes up to 8 attempts to reach participants for each scheduled call, beginning on the scheduled call day and proceeding with 2 attempts per day for 4 days or until the call is completed.

Each IVR call assesses a participant’s smoking status, current use of smoking cessation medication, medication side effects, and offers the option of connection to live telephone counseling support. The automated call delivers motivational messages specifically tailored to the participant’s progress in his or her quit attempt. For example, smokers who failed to use medication after discharge hear, “Using medicine doubles your chance of quitting smoking for good. We recommend that you talk with our tobacco counselor about how to get started on your medicine”.

Any participant can request to speak with a smoking cessation counselor during any automated IVR call. Additionally, the IVR system specifically recommends that participants whose responses meet the pre-specified criteria speak to a live counselor. Criteria that trigger the system to recommend a transfer include the following: (1) participants who have not started their smoking cessation medication after discharge or prematurely stopped using pharmacotherapy prior to a 90-day course; (2) participants who returned to smoking after discharge but still wish to quit; (3) participants who are quit but have low confidence in their ability to remain abstinent; (4) participants who report having medication side effects; and (5) participants who are due for a medication refill.

#### Access to smoking cessation telephone counseling support

An innovation of the HH2 study is the facilitation of real-time transfer from the automated call to a live telephone counselor in order to maximize treatment continuity at the time of need. This immediate transfer is made possible through coordination between the IVR provider, TelASK Technologies (Ottawa, Canada), and the telephone counseling provider, Alere Wellbeing Inc., (Seattle, WA) (Table 1). Alere offers its standard protocol of up to 5 proactive telephone counseling calls over 3 months to support a quit attempt [27]. The goal is to have a seamless link between IVR monitoring and request for telephone counseling support. Alere staff also provide smokers with NRT refills when these are due, thereby facilitating medication adherence.

Upon transfer from the IVR system to the quitline provider, the participant speaks to a registration agent who collects basic information and then routes the caller to a smoking cessation counselor (“Quit Coach”). The Quit Coach provides the following: (1) an introduction, presenting an overview of topics and the purpose of the call; (2) assessment of the participant’s smoking history, confidence and motivation to quit; (3) specific insight for the participant—summarizing the participant’s strengths, weaknesses, and barriers to quitting; (4) an action plan, which consists of setting a realistic quit date, brainstorming strategies to cope with urges, planning to rehearse coping skills, and addressing medication use, environmental factors, and social support; and (5) schedule for the next call, tailored to the participant’s quit date. The Quit Coach calls the participant for up to 4 additional counseling sessions. Call 2 is scheduled on or around the quit date and includes management of withdrawal symptoms, appropriate use of medications, strategies to maintain abstinence in high-risk situations, and early relapse prevention. Calls 3 through 5 focus on relapse prevention and addressing participant concerns and questions [28]. In addition, once registered with Alere, a participant can call in to speak with a Quit Coach directly at any time.

#### Pharmacotherapy

A free 1-month supply of FDA-approved smoking cessation pharmacotherapy is delivered to the participant prior to discharge. Cessation medication options include NRT (nicotine patches, lozenges, or gum), bupropion, or varenicline. Either a single medication, or more commonly, a combination of medications is prescribed (e.g., nicotine patch and lozenge). The participant chooses the pharmacotherapy, guided by the hospital tobacco counselor, who considers the participants’ number of cigarettes smoked daily, time to smoking first cigarette in the morning, participant preference, past cessation medication experience, and medical contraindications. The hospital physician writes the prescription which is filled by the hospital pharmacy or, at NSMC, a community pharmacy. All participants receive a handout with information on proper medication use, side effects, and instructions on how to obtain the two additional months of free medications through the IVR, quitline, and study staff. On rare occasions when a participant is discharged before receiving the prescribed cessation medication in hand, the research staff ships the medication using overnight delivery and calls the participant to ensure receipt of the medication.

When a participant requests a free refill of NRT, the Quit Coach asks about current medication use, reviews medication prescribed upon discharge, revisits exclusion questions, and refills the medication. An automatic delivery system from Alere’s warehouse delivers the medication within 48-72 hours. To refill a prescription-only cessation medication, a Quit Coach notifies a member of the research staff, and the study staff assesses medication use and side effects with the participant. Subsequently the research staff arranges to have the refill shipped from the pharmacy through which it was originally filled (Figure 1).

#### Standard care

Participants assigned to the Standard Care group receive the same bedside counseling session in the hospital as the Sustained Care group does. The counselor informs smokers about post-discharge counseling resources, provides specific advice to call the state telephone quitline, makes a specific recommendation to the hospital physician for post-discharge medication, and completes a consultation note in the participant’s hospital record. No additional resources are provided to the participant after discharge from the hospital.

### Assessments

At baseline a survey is administered by research staff immediately after informed consent is obtained. At 1, 3, and 6 months after hospital discharge, research staff contact participants by telephone to conduct follow-up surveys. Participants who report being continuously abstinent from tobacco for 6 months are re-contacted at 12 months.

Prior to each follow-up call, participants are sent a postcard reminding them that research staff will be contacting them in the near future. The research staff makes up to 15 call attempts, calling at different times and on different days, making more attempts during the participants’ self-reported preferred call time. After an 8^th^ unsuccessful call attempt, the research staff calls the participants’ alternate contacts, people whom the participants specified would know how to reach them, to verify the participants’ contact information. Mailed surveys with a stamped return envelope are sent upon reaching the 8^th^ and 15^th^ unsuccessful call attempts. Upon reaching a 10^th^ unsuccessful call attempt, the participant receives a text message requesting a call to the study hotline. Participants are compensated $20 for completing each follow-up survey.

At the 6 month follow-up, a participant’s self-report of past 7-day tobacco abstinence is biochemically confirmed. The participant is asked to mail a saliva sample for assay of cotinine, a nicotine metabolite with a 16-hour half-life [29]. Because this test is falsely positive for participants using NRT or electronic cigarettes (e-cigarettes), we request that individuals using either of these visit the hospital for an expired air carbon monoxide (CO) test. Participants receive $50 dollars for returning the cotinine kit or completing a CO test, regardless of the result obtained. Because smoking status can change between self-report and the time of saliva collection, we reassess participants’ use of any form of nicotine in the 7 days prior to the date of saliva collection or CO test. This includes use of any tobacco products, e-cigarettes, and nicotine replacement products.

## Measures

### Participant surveys

The survey instruments cover the following domains (Table 3).

**Table 3:** **Helping HAND trial measures and schedule of administration**

	**Baseline**	**Outcomes**			
**Measure**		**1 Month**	**3 Month**	**6 Month**	**12 Month**
Demographic factors					
Age, sex, race/ethnicity, educational attainment, employment status, martial/partner status, type of housing	X				
Smoking history					
Number of cigarettes per day, use of other tobacco products, use of electronic-cigarette, number of years smoking, prior quit attempt, smoker in household, home smoking policy	X				
Nicotine dependence (time to first cigarette)	X				
***Previous cessation medication use***	***X***				
Importance in ability to quit	X				
Confidence in ability to quit/stay quit	***X***	X	***X***	***X***	
Tobacco abstinence status–self-report					
Cigarette smoking status	X	X	X	X	***X*** ^1^
Non-cigarette tobacco use status	X	X	X	X	***X*** ^1^
Electronic-cigarette use	X	X	X	X	***X*** ^1^
Tobacco abstinence status–biochemical validation					
Saliva cotinine or expired aired CO				X	
Quit attempt (>24 hours)	X	X			
Hospital admission or emergency room visit		X	X	X	
***Quality of life (EQ-5D)***	***X***			***X***	
***Anxiety and depression symptoms (PHQ-4)***	***X***	***X***	***X***	***X***	
Alcohol use (AUDIT-C)	X			***X***	
***Other non-tobacco substance use***	***X***				
Satisfaction with IVR		X	***X***		
***Satisfaction with Quit Coach***		***X***	***X***		
Smoking during index hospital stay		X			
Smoking cessation medication used after discharge		X	X	X	
Smoking cessation counseling used after discharge		X	X	X	
***Other smoking cessation programs/resources used***		***X***	***X***	***X***	

***Items in bold italics were only in Helping HAND 2.***

All other measures were used in both Helping HAND 1 and 2.

^1^Asked only of participants who self reported continuous abstinence at 6 month follow-up.

*Demographic factors*: sex, age, race/ethnicity, educational attainment, employment status, marital/partner status, and type of housing are collected by participant interview and chart review at baseline.

*Tobacco use history* includes cigarettes per day, use of other tobacco products, and use of electronic cigarettes in the month prior to admission, number of years smoking, prior quit attempt (yes/no), presence of a smoker in the household, and home smoking policy. *Prior tobacco treatment* is assessed at baseline by asking about previous use of NRT, bupropion, varenicline, telephone counseling support, and in-person counseling support. *Nicotine dependence* is assessed by asking time to first cigarette of the day.

*Intention to quit* upon discharge is assessed with the item described in Table 2. Perceived *importance* of quitting and perceived *confidence* in ability to quit are assessed with 5-point Likert-type scales ranging from “not at all” to “very” at baseline. Confidence is reassessed at each follow-up.

*Alcohol use* in the past year is assessed at baseline and 6 months using the three-item Alcohol Use Disorder Identification Test—Consumption [30]. *Other substance use* is assessed at baseline by asking about any use in the past year of marijuana, cocaine, crack, stimulants, and opioids. Injection drug use is assessed by asking about participants’ lifetime use and their use within the past year.

*Anxiety and depression symptoms* are assessed at baseline and at each follow-up using the Patient Health Quessionaire-4, a 4-item inventory rated on a 4-point Likert scale ranging from “not at all” to “nearly every day” [31]. The first two questions measure anxiety symptoms and the latter two measure depression symptoms. Scores range from 0-12, with higher scores indicating more symptoms.

*Quality of life* is measured at baseline and 6-month follow-up using the EQ-5D instrument [32]. It assesses quality of life on a 5-point Likert scale with answers ranging from “no problems” to “unable” and “not at all” to “extremely”. The questions range from physical mobility (problems walking, washing, dressing, pain, and discomfort) to psychological (feeling anxious or depressed).

#### Inpatient experience with tobacco abstinence

At 1-month follow-up, participants rate the difficulty they had maintaining abstinence in the hospital by answering the question, “How hard was it not to smoke while you were in the hospital?” (4-point Likert-type scale, “not at all” to “very”). Participants are asked whether they used any FDA-approved smoking cessation medications in the hospital. Cigarette use and electronic cigarette use while in the hospital are assessed by asking, “Did you smoke a cigarette (or an electronic cigarette), even a puff, during your stay in the hospital?” Participants who report using either product are asked, “Did you smoke inside the hospital building, outside, or both?”

### Hospital record review

We obtain health insurance, hospital length of stay, primary and secondary hospital discharge diagnoses, hospital service, and participants’ disposition after discharge (home or rehabilitation facility) from hospital records. Chart review at baseline is done to obtain height, weight, and history of co-morbid medical diagnoses; these include hypertension, diabetes, dyslipidemia, myocardial infarction or coronary heart disease, stroke, cancer (other than skin), and emphysema or chronic bronchitis.

### Outcome measures

#### Tobacco abstinence

The primary smoking outcome is a biochemically-validated 7-day point prevalence abstinence from all tobacco products at 6 months after discharge. This is determined by responses to these questions: “In the past 7 days have you smoked a cigarette, even a puff?” and “In the past 7 days, have you used any tobacco products other than cigarettes such as cigars, pipes, snus, or chew?” Participants are considered to be smokers for the primary outcome if they are lost to follow-up or report tobacco abstinence but do not provide a biochemical sample that meets criteria for abstinence (saliva cotinine ≤10 ng/ml or expired air CO ≤9 ppm).

Secondary outcome measures of tobacco abstinence status include the following: (1) self-reported 7-day point prevalence tobacco abstinence at 1, 3, and 6 months; (2) self-reported sustained 7-day abstinence from cigarettes and other tobacco products at 1, 3, and 6 months; (3), self-reported continuous abstinence from cigarettes and other tobacco products at 1, 3, 6, and 12 months (‘Since you left the hospital, have you smoked a cigarette, even a puff? Have you used any other tobacco products such as cigars, pipes, snus, or chew?)’; (4) duration of tobacco abstinence after discharge (How soon after you left the hospital, did you smoke your first cigarette, even a puff?); and (5) proportion of participants who make a 24-hour quit attempt after discharge (‘Since you left the hospital have you not smoked for 24 hours because you were trying to quit?’).

Whether to classify e-cigarette users as tobacco abstinent or not is under discussion by tobacco researchers. No clear criteria have yet been adopted. For this study, we consider e-cigarette use to be comparable to long-term NRT use. Individuals who report abstinence to all conventional tobacco products but are using e-cigarettes and meet criteria for biochemical validation of abstinence by expired air CO are considered to be tobacco abstinent. A sensitivity analysis will be done to determine how tobacco abstinence rates change when e-cigarettes only users are classified as tobacco users.

#### Smoking cessation treatment use and adherence

We hypothesize that the intervention is mediated by increasing participants’ use of evidence-based pharmacotherapy and counseling after discharge. Participants report on the extent of any use of either modality at each outcome assessment. Smoking cessation counseling is defined as telephone or in-person counseling from any source including a physician. Pharmacotherapy includes use of NRT (nicotine patch, gum, lozenge, inhaler, or nasal spray), bupropion, or varenicline. For medications used, we assess dose, starting date of use, frequency and duration of use, reason for termination, and whether the patient changed their dosage due to side effects.

The principal measure of treatment use is the use of any pharmacotherapy or counseling after discharge; we will also calculate the proportion of participants who use pharmacotherapy and counseling separately. Outcome measures for pharmacotherapy include any use and duration of use. Outcome measures for counseling include any use and number of contacts.

#### Health care utilization

We will calculate the rate of all-cause re-hospitalizations, re-hospitalization for coronary heart disease, emergency room visits, and all-cause deaths over 6 months after the index hospitalization. Measures include (1) number of hospital readmissions, (2) length of stay in hospital, (3) number of emergency room visits, and (4) total number of hospitalizations and emergency room visits. An electronic health record tracking system automatically notifies research staff when a study participant has an emergency room visit or hospitalization at facilities in Partners Healthcare System, the integrated health care delivery system to which MGH and NSMC belong. At the end of the study, data on these events will be obtained from Partners billing data. At UPMC, information about hospital readmissions to UPMC-affiliated hospitals is obtained from chart review of participants’ electronic health records.

Information about hospitalizations and emergency room visits outside UPMC and Partner’s Healthcare System are obtained from participant surveys, by asking the questions “Since you were discharged from [hospital name] on [date] have you gone to the emergency room or stayed overnight at any hospital? This includes rehabilitation or skilled nursing facilities”. We request hospital name, type of visit (either ED, admission, inpatient rehab,) length of stay, and the hospitalization date. This is supplemented by requesting medical records of admissions to hospitals outside Partners HealthCare System and UPMC-affiliates.

#### Program satisfaction

Participants in the Sustained Care arm are asked about their satisfaction with the telephone counseling by the Quit Coaches and the automated IVR calls. Participants who ever spoke to a Quit Coach are asked: (1) “How helpful was the Quit Coach?” (“not at all” to “very”); (2) “If a friend or family member who smokes and wanted to stop were hospitalized, would you recommend that they speak with a Quit Coach to help them stop smoking?” (“strongly recommend” to “strongly not recommend”); and (3) an open-ended question, “What, if anything, was helpful or not helpful about the Quit Coach program?” Those who received at least one IVR call are asked parallel questions regarding the automated telephone system, using the same scales. Additionally we ask, “How helpful was it to have the automated telephone calls refill your stop smoking medication for you?” (“not at all” to “very”).

### Quality assurance

Multiple actions are taken to ensure the consistency of TTS services, recruitment, and enrollment across all three sites. These included creating standardized protocols across sites for counseling, referring, and enrolling patients. All counselors and recruiters were trained together prior to recruitment, specifically focusing on inclusion and exclusion criteria. Regular meetings of certified TTS counselors from all sites are held to ensure that the same standardized protocol is followed for referrals. Follow-up assessments were centralized at one study site.

### Data analysis

A total sample of 1350 was planned to detect a difference of 4.8% (12.8% vs. 8.0%) in the primary outcome measure, validated 7-day tobacco abstinence at 6-month follow-up, with 80% power and a 2-tailed type I error rate of .05. Analysis of study data will use an intention-to-treat approach. In the primary analysis, subjects lost to follow-up due to all circumstances will be coded as smokers for smoking status outcomes. A sensitivity analysis will be conducted based on different assumptions on missing data [33]. Multiple imputation techniques will also be used to take into account uncertainty due to missing data.

#### Primary outcome

The primary smoking outcome is biochemically-validated 7-day point prevalence abstinence from all tobacco products at 6 month follow-up. For the analysis, Cochran-Mantel-Haenzel tests will first be used to determine whether intervention effects are consistent across the 3 study sites. Logistic regression analyses will be used to compare the effect of study arm on the primary outcome measure, adjusting for study site.

#### Secondary outcomes

For all outcomes measured at 1, 3, and 6 months, cross-sectional analyses will be used to compare between study groups. Secondarily, a longitudinal analysis using Generalized Estimating Equations (GEE) techniques will be conducted to assess the overall impact of Sustained Care by including data from all follow-up times. Similar to the primary outcome, regression models will be used to compare treatment group adjusting for site and any important confounders; these will be logistic regression for dichotomized outcomes (e.g. self-reported abstinence, any counseling/medication use), and Poisson regression for the number of event outcomes (e.g. number of counseling contacts, number of emergency room visits, number of hospital readmissions). Survival analysis techniques will be used to analyze the duration-related outcome variables (self-reported duration of continuous abstinence and duration of post-discharge medication use). Kaplan-Meier curves will be used to estimate the median duration of tobacco abstinence and medication use. Cox proportional hazard models will examine intervention effects adjusting for potential confounding factors.

*Cost-effectiveness* will be assessed using cost per quit as the main outcome in order to compare our results with other published studies [22,34,35]. The incremental costs per quit are estimated as follows: (Total costs of Sustained Care – Total costs for Standard Care)/(Total successful quits at six months for Sustained Care – Total successful quits at six months for Standard Care) where successful quits are defined according to our primary outcome of 7-day point prevalence abstinence. The major costs tracked in the study are as follows: (1) initial counselor assessment pre-discharge, which includes counselor chart review, counseling, and medication recommendations; (2) Quit Coach medication assessment and cessation support post-discharge; (3) IVR service; and (4) medication costs, including dispensing and delivery. For both study arms, we will track the portion of counseling and medication costs paid for by participants out of pocket and/or by insurance.

## Trial status

A total of 1,359 participants were enrolled in the study between December 2012 and July 2014. The follow-up of these participants is in progress. Enrollment data from the HH2 study can be compared to enrollment data from the HH1 study to assess whether the HH2 study met its goal of enrolling a higher proportion of hospitalized smokers by broadening the eligibility criteria to permit enrollment of smokers with comorbid alcohol or other substance abuse. In the HH2 study, 1416 (72%) of the 1959 smokers who were fully screened met eligibility criteria and 1359 (69%) were enrolled. By comparison, in the HH1 study, only 432 (48%) of the 904 smokers who were fully screened met eligibility criteria and only 397 (44%) enrolled. In the HH2 study only 23 (1%) of 1959 patients were excluded for alcohol or other substance abuse, compared to 254 patients (28%) of 904 patients excluded for these reasons in the HH1 study. The HH2 study sample also included a higher proportion of smokers with HIV infection (4.5% [n = 61] vs. 1% [n = 4] in the HH1 study). Thus, relaxing the restriction on enrolling smokers with non-tobacco substance abuse in the HH2 study produced a sample that was more representative of all hospitalized smokers than had been recruited into the HH1 study.

## Discussion

The Helping HAND 2 trial tests whether a coordinated system to sustain smoking cessation treatment initiated during a hospitalization increases the proportion of hospitalized smokers who are tobacco abstinent 6 months after discharge. The program aims to achieve this by removing barriers to continued use of smoking cessation medication and counseling. It resembles the intervention that was effective in the single-site HH1 trial [12] but extends this earlier work. It tests the intervention’s effectiveness in three hospitals in two states, and the intervention provides a new real-time direct transfer from the automated call to a live smoking cessation counselor when smokers request additional assistance. HH2 also tests the intervention in a more medically and socially diverse sample that includes smokers with other substance abuse or HIV infection, vulnerable populations whose smoking prevalence exceed that of the general population. The study enrollment data demonstrate that the HH2 study succeeded in its goal of enrolling a larger proportion of smokers admitted to the study hospitals and including smokers in more vulnerable populations. Analysis of outcome data, which is still being collected, will determine whether the intervention will retain its effectiveness. If effective, this model could be adopted by U.S. hospitals to help reduce population smoking rates, thereby decreasing tobacco-related mortality, morbidity, and health care costs.

## Abbreviations

Helping HAND: Helping Hospital-initiated Assistance for Nicotine Dependence study
HH1: Helping HAND 1
HH2: Helping HAND 2
NRT: Nicotine replacement therapy
IVR: interactive voice response
TTS: Tobacco Treatment Service
e-cigarette: electronic-cigarette
CO: carbon monoxide
MGH: Massachusetts General Hospital
NSMC: North Shore Medical Center
UPMC: University of Pittsburgh Medical Center
